# Atlanta Medical College

**Published:** 1858-06

**Authors:** 


### Atlanta Medical College.

To those who feel interested in the welfare of the Atlanta Medical College, we would remark, that its prospects were never so flattering, either in the number or character of the class, the size of which will very considerably exceed that of last year; to the full extent we are confident of the regular annual increase in the numbers of those who have heretofore attended this Institution.

This regular and decided advance would be gratifying under any circumstances, but it is especially so, at a time when the country has just been passing through the recent terrible financial crisis, from which indeed, it has not yet recovered; and when the medical schools, almost without an exception, are showing diminished numbers.

